# Supplementary material for: Mendelian Randomization Study on Amino Acid Metabolism Suggests Tyrosine as Causal Trait for Type 2 Diabetes
Source: Nutrients. 2020 Dec 19;12(12):3890. doi: 10.3390/nu12123890 (PMC7766372; doi:10.3390/nu12123890)
Supplement: Supplementary file 1 [file nutrients-12-03890-s001.zip › Supplement s1/Supplement A.pdf]

Mendelian Randomization study on amino acid metabolism suggests tyrosine as causal trait for type 2 diabetes

Susanne Jäger[1,2], Rafael Cuadrat[1,2], Clemens Wittenbecher[1,2,3], Anna Floegel[4], Per Hoffmann[5,6], Cornelia Prehn[7], Jerzy Adamski[2,7,8,9], Tobias Pischon[10,11,12], Matthias B. Schulze[1,2,13*]

[1] Department of Molecular Epidemiology, German Institute of Human Nutrition Potsdam-Rehbruecke, Nuthetal, Germany.

[2] German Center for Diabetes Research (DZD), Germany.

[3] Department of Nutrition, Harvard T.H. Chan School of Public Health, Boston, MA, United States.

[4] Leibniz Institute for Prevention Research and Epidemiology-BIPS, Bremen, Germany.

[5] Human Genomics Research Group, Department of Biomedicine, University of Basel, Basel, Switzerland.

[6] Institute of Human Genetics, Division of Genomics, Life & Brain Research Centre, University Hospital of Bonn, Bonn, Germany.

[7] Research Unit Molecular Endocrinology and Metabolism, Helmholtz Zentrum München, German Research Center for Environmental Health, Neuherberg, Germany.

[8] Chair of Experimental Genetics, Center of Life and Food Sciences Weihenstephan, Technische Universität München, Freising-Weihenstephan, Germany.

[9] Department of Biochemistry, Yong Loo Lin School of Medicine, National University of Singapore, 8 Medical Drive, Singapore 117597, Singapore

[10] Molecular Epidemiology Research Group, Max Delbrueck Center for Molecular Medicine in the Helmholtz Association (MDC), Berlin, Germany.

[11] Charité – Universitätsmedizin Berlin, corporate member of Freie Universität Berlin, Humboldt-Universität zu Berlin and Berlin Institute of Health (BIH), Berlin, Germany

[12] MDC/BIH Biobank, Max Delbrueck Center for Molecular Medicine in the Helmholtz Association (MDC) and Berlin Institute of Health (BIH), Berlin, Germany

[13] Institute of Nutritional Science, University of Potsdam, Potsdam, Germany.

Supplemental Tables

Table S1 Characteristics of studies included within the DIAGRAM consortium

Table S2 Associations of single genetic instruments used for MR of amino acids traits from EPIC-Potsdam and type 2 diabetes

Table S3 Total causal effects of amino acid traits from EPIC-Potsdam and of type 2 diabetes using independent instruments ($R^2<0.001$)

Table S4 Total causal effects of amino acid traits from Shin et al. and type 2 diabetes and sensitivity analyses

Table S5 Associations of single genetic instruments used for MR of amino acids traits from Shin et al. and type 2 diabetes

Table S6 Direct causal effects of diabetes associated amino acid traits from Shin et al. and type 2 diabetes

Table S7 Associations of single genetic instruments used for MVMR of amino acids traits from Shin et al. and type 2 diabetes

Table S8 Total causal effects of tyrosine risk of type 2 diabetes using other public data sources

Table S9 Associations of single genetic instruments used for MR of tyrosine from Kettunen et al. and Locke et al. and type 2 diabetes

Table S10 Total causal effects of tyrosine from Shin et al. and other risk factors for type 2 diabetes

Table S11 Instruments from genetic risk score for insulin resistance

Table S12 Total causal effects of insulin resistance on amino acid traits from EPIC-Potsdam

Table S13 Associations of single genetic instruments used for MR of insulin resistance and amino acids traits from EPIC-Potsdam

Table S14 Total causal effects of insulin resistance and single amino acids

Table S15 Associations of single genetic instruments used for MR of insulin resistance and single amino acids

Supplemental Figures

Figure S1 Network structure of amino acids within EPIC-Potsdam

Figure S2 Flow-chart of final study population in EPIC-Potsdam

Figure S3 Example flow charts of inclusion and exclusion of SNPs into the analysis

Figure S4 Manhattan plots visualizing GWAS results for amino acid traits in EPIC-Potsdam

Table S1 Characteristics of studies included within the DIAGRAM consortium

See Excel file in Supplement B

Previously published in Mahajan et al. 2018 [1]

Table S2 Associations of single genetic instruments used for MR of amino acids traits from EPIC-Potsdam and type 2 diabetes

See Excel file in Supplement B

Table S3 Total causal effects of amino acid traits from EPIC-Potsdam and of type 2 diabetes using independent instruments (R^2<0.001)

Amino acid trait	Instruments	N (SNPs)*	Beta (SE) from IVW	P-Value	Heterogeneity between SNPs; Q-statistic, p-value	Directional horizontal pleiotropy **; Egger-intercept (SE), p-value	Outlier detected
**Glycine**	suggestive	2/2	-0.006 (0.011)	0.621	no; 0.16, 0.68	n.a.	no
	genome-wide	1/1	-0.007 (0.011)	0.547	n.a	n.a	n.a
**Glycine/Serine**	suggestive	4/4	0.018 (0.041)	0.663	yes; 37.0, <0.001	no; 0.04 (0.02), 0.16	n.a
	genome-wide	2/2	-0.007 (0.013)	0.588	no; 0.07, 0.79	n.a.	n.a.
**Phenylalanine**	suggestive	1/1	0.053 (0.045)	0.237	n.a.	n.a.	n.a.
**Phenylalanine/Arginine**	suggestive	2/2	0.043 (0.029)	0.131	no; 0.39, 0.53	n.a.	no
**Serine/Phenylalanine**	suggestive	3/4	0.000 (0.023)	0.995	no; 0.61, 0.74	no; 0.08 (0.16), 0.62	n.a.
**Tryptophan/Glutamine**	suggestive	2/2	0.003 (0.029)	0.931	no; 0.17, 0.68	n.a.	n.a.
**Tyrosine/Methionine**	suggestive	3/3	-0.024 (0.023)	0.296	yes; 26.5, <0.001	no; 0.10 (0.1), 0.33	n.a.
	genome-wide	1/1	-0.141 (0.033)	<0.001	n.a.	n.a.	n.a.
**xLeucine/Methionine**	suggestive	1/2	-0.031 (0.043)	0.437	n.a.	n.a.	n.a.
**Valine/xLeucine**	suggestive	1/1	-0.012 (0.044)	0.777	n.a.	n.a.	n.a.

IVW, inverse variance weighted method; n.a., not applicable; SE, standard error; T2DM, type 2 diabetes
* used instruments/suitable instruments available in the GWAS of T2DM [2]
** assessed by MR-Egger (>2 variants needed)

Table S4 Total causal effects of amino acid traits from Shin et al. and type 2 diabetes and sensitivity analyses

Amino acid trait*	N (SNPs)**	Beta (SE) from IVW/Wald ratio method for T2DM*	P-Value	Heterogeneity Q-statistic, p-value	Directional horizontal pleiotropy*** Egger-intercept (SE), p-value
Glycine	1	-0.05 (0.09)	0.61	n.a.	n.a.
Phenylalanine	1	1.28 (0.87)	0.14	n.a.	n.a.
Tryptophan	17	-0.15 (0.47)	0.74	yes 40.1, 0.0008	0.04 (0.03), 0.19
Tyrosine	3	-1.53 (0.57)	0.008	no 4.5, 0.10	0.07 (0.05), 0.40
Methionine	1	-1.17 (1.00)	0.24	n.a.	n.a.
Valine	1	2.47 (0.77)	0.001	n.a.	n.a.
Leucine	12	0.98 (0.45)	0.03	No 19.7, 0.05	-0.02 (0.01), 0.17

IVW, inverse variance weighted method; n.a., not applicable; SE, standard error; T2DM, type 2 diabetes
* assessed using public data by Shin et al. [3] and Mahajan et al. [2]

** significance threshold = 5×10^{-8} ; clumping threshold R^2=0.001

*** assessed by MR-Egger (>2 variants needed)

Tryptophan	Tyrosine	Leucine

MR-results

Tryptophan

method	nsnp	b	se	pval
MR Egger	17	-8.2857507	5.8987919	0.1804935
Weighted median	17	0.0767884	0.5021401	0.8784597
Inverse variance weighted	17	-0.1544658	0.4667298	0.7406798
Simple mode	17	-0.0037496	1.0977672	0.9973170
Weighted mode	17	-0.0037496	1.0323380	0.9971469

Tyrosine

method	nsnp	b	se	pval
MR Egger	3	-8.2403783	4.8250444	0.3372282
Weighted median	3	-1.3442285	0.5786362	0.0201739
Inverse variance weighted	3	-1.5295305	0.5726261	0.0075607
Simple mode	3	-1.1752509	0.8038651	0.2812442
Weighted mode	3	-0.9697738	0.8946540	0.3916622

Leucine

method	nsnp	b	se	pval
MR Egger	12	4.8884133	2.6701272	0.0970520
Weighted median	12	0.6547975	0.5200194	0.2079658
Inverse variance weighted	12	0.9763795	0.4511942	0.0304652
Simple mode	12	0.4258693	0.8254132	0.6161036
Weighted mode	12	0.4258693	0.9088668	0.6485243

Forest plots

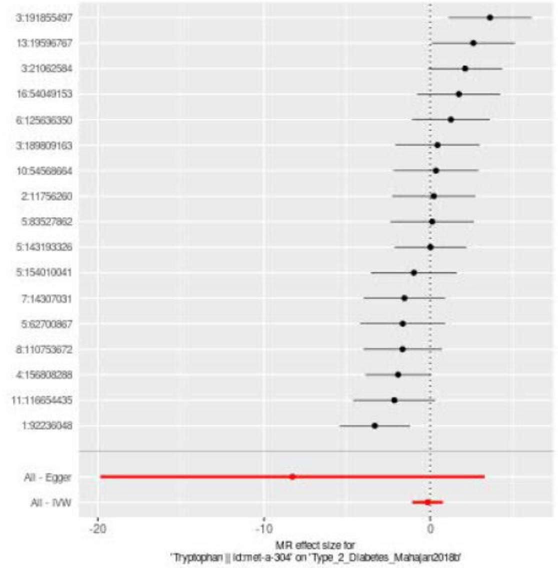

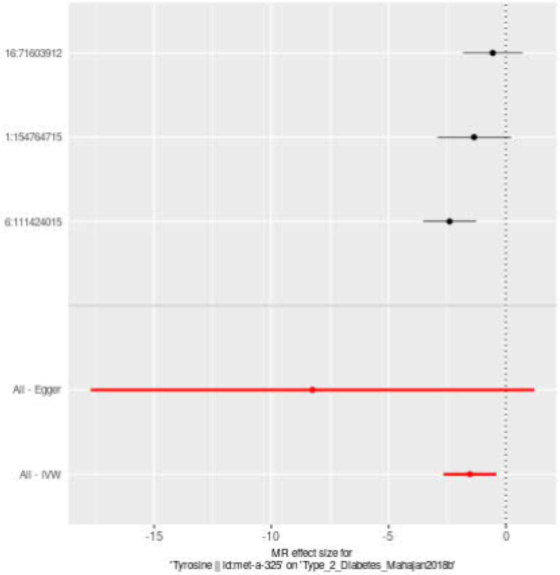

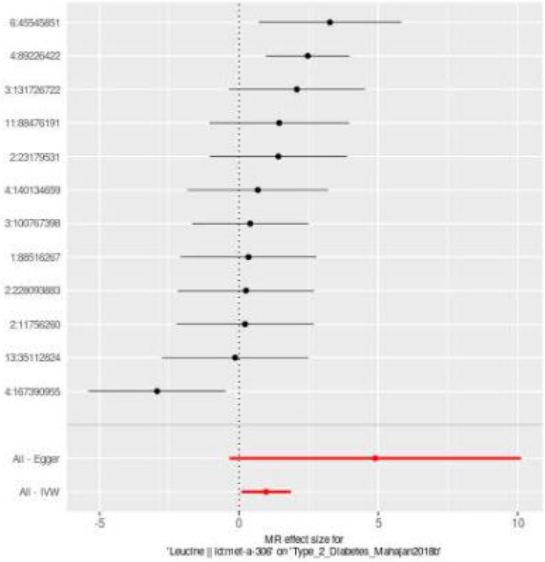

<table>
<tr><th>Tryptophan</th><th>Tyrosine</th><th>Leucine</th></tr>
<tr><td colspan="3">Funnel plots</td></tr>
<tr>
<td>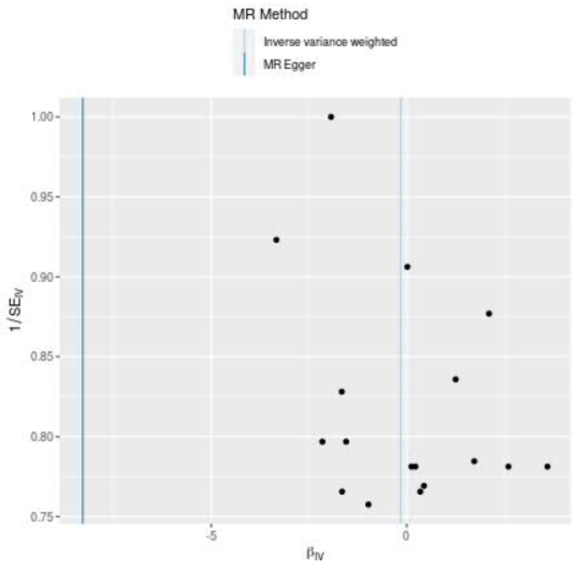
</td>
<td>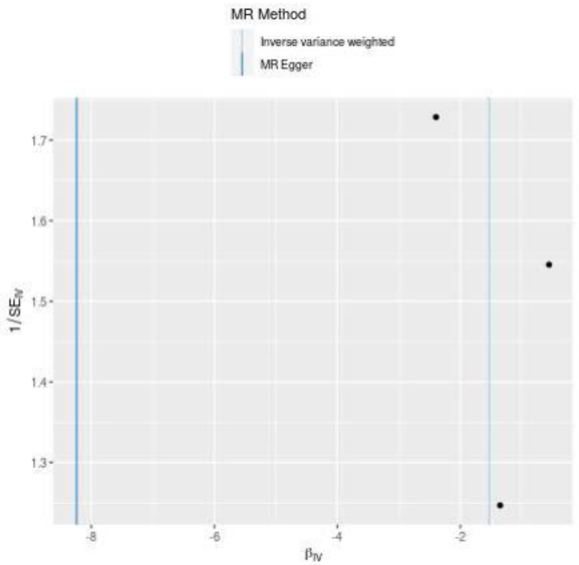
</td>
<td>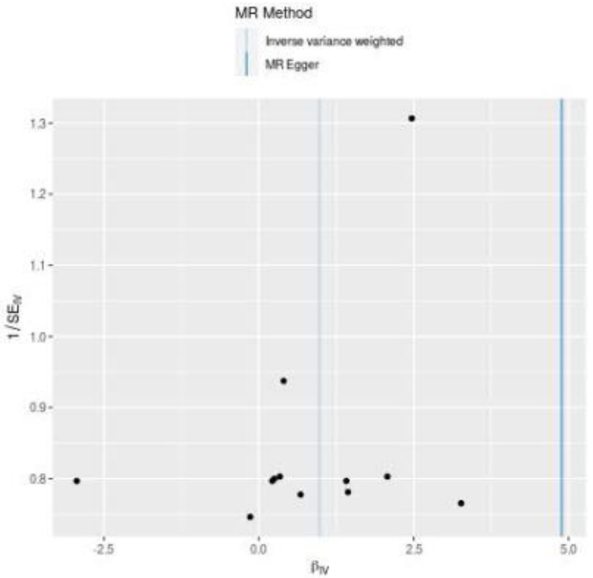
</td>
</tr>
<tr><td colspan="3"></td></tr>
</table>

Tryptophan	Tyrosine	Leucine
**Leave-one-out sensitivity analysis**		

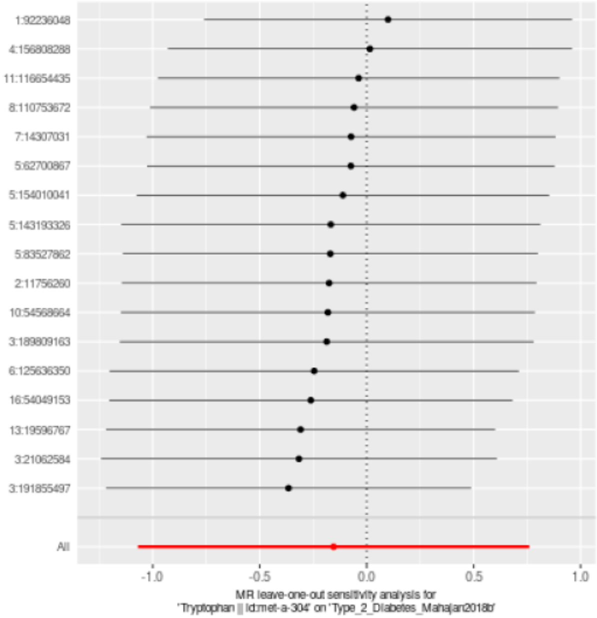

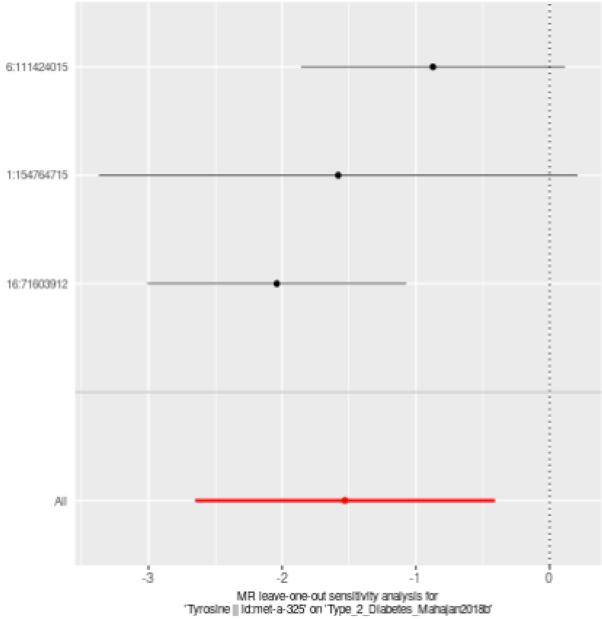

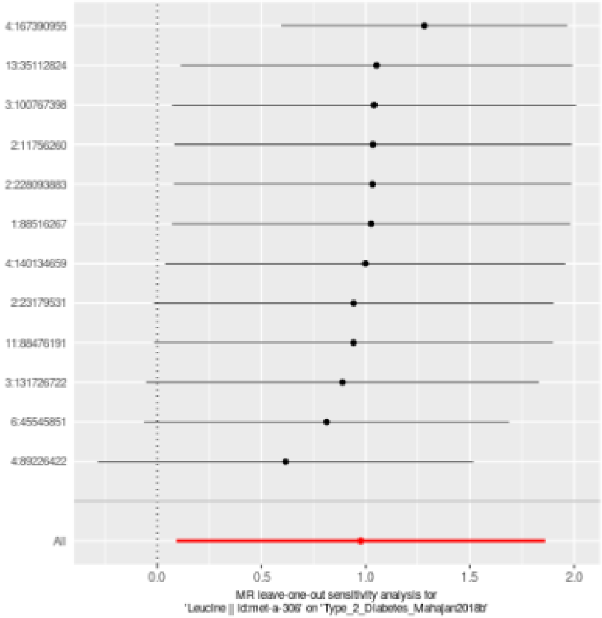

Table S5 Associations of single genetic instruments used for MR of amino acids traits from Shin et al. and type 2 diabetes

See Excel file in Supplement B

Table S6 Direct causal effects of diabetes associated amino acid traits from Shin et al. and type 2 diabetes

Amino acid trait*	N(SNPs)**	Beta (SE) from multivariable IVW method for T2DM*	P-Value	Conditional F-statistic***	Beta (95% CI) **** accounting for weak instruments
Glycine	1	0.05 (0.16)	0.77	0.37	0.04 (-0.79-1.97)
Phenylalanine	1	1.07 (1.30)	0.41	0.53	1.19 (-2.31-4.46)
Tryptophan	17	-0.20 (0.70)	0.77	2.02	-0.17 (-1.81-1.51)
Tyrosine	3	-1.66 (0.78)	0.03	0.36	-1.76 (-3.68--0.03)
Leucine	11	-0.70 (1.56)	0.65	0.31	-0.66 (-4.62-2.40)
Isoleucine	0	1.21 (2.27)	0.59	0.08	1.23 (-2.51-5.07)
Valine	1	1.33 (2.54)	0.60	0.10	1.19 (-2.60-6.96)

* assessed using public data by Shin et al. [3] and Mahajan et al. [2]

** significance threshold = 5×10^{-8}; clumping threshold R^2=0.001

*** using covariance matrix from EPIC-Potsdam phenotypes

**** Confidence intervals are calculated using a non-parametric bootstrap with 1000 iterations

Table S7Associations of single genetic instruments used for MVMR of amino acids traits from Shin et al. and type 2 diabetes

See Excel file in Supplement B

Table S8 Total causal effects of tyrosine risk of type 2 diabetes using other public data sources

Tyrosine from Kettunen et al. 2016; n=24,925

method	nsnp	b	se	pval
MR Egger	3	-0.4225385	0.2266672	0.3134551
Weighted median	3	-0.1176278	0.0586697	0.0449720
Inverse variance weighted	3	-0.0951712	0.0611713	0.1197518
Simple mode	3	-0.1142686	0.0897026	0.3307251
Weighted mode	3	-0.1366342	0.0614610	0.1562546

Tyrosine from Locke et al. 2019; n=8,754

method	nsnp	b	se	pval
MR Egger	3	0.2561767	0.2200496	0.4517975
Weighted median	3	0.1617589	0.0420126	0.0001180
Inverse variance weighted	3	0.1587883	0.0499333	0.0014727
Simple mode	3	0.1900944	0.0591888	0.0847988
Weighted mode	3	0.1783170	0.0552536	0.0840818

Forest plots

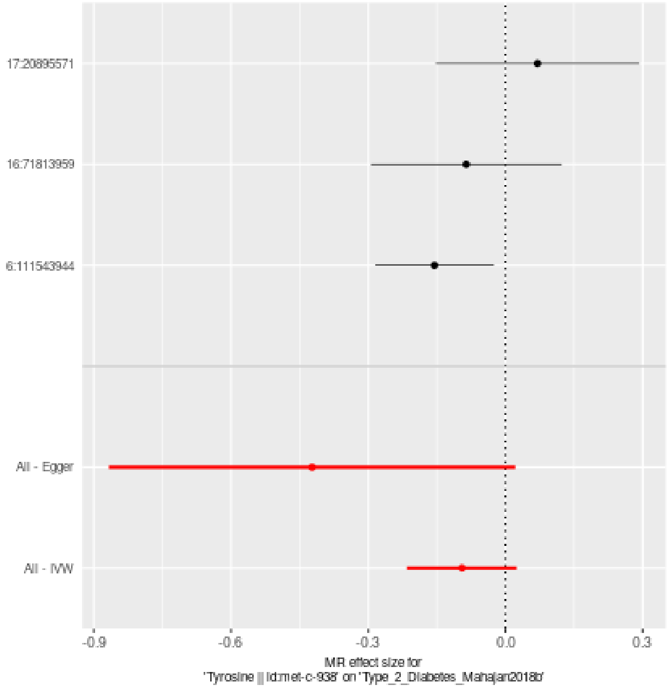

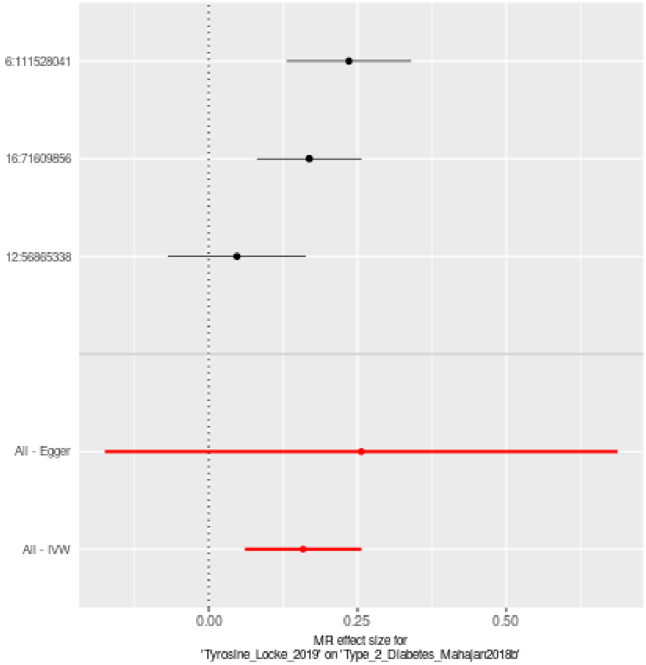

Tyrosine from Kettunen et al. 2016; n=24,925	Tyrosine from Locke et al. 2019; n=8,754
**Funnel plots**	
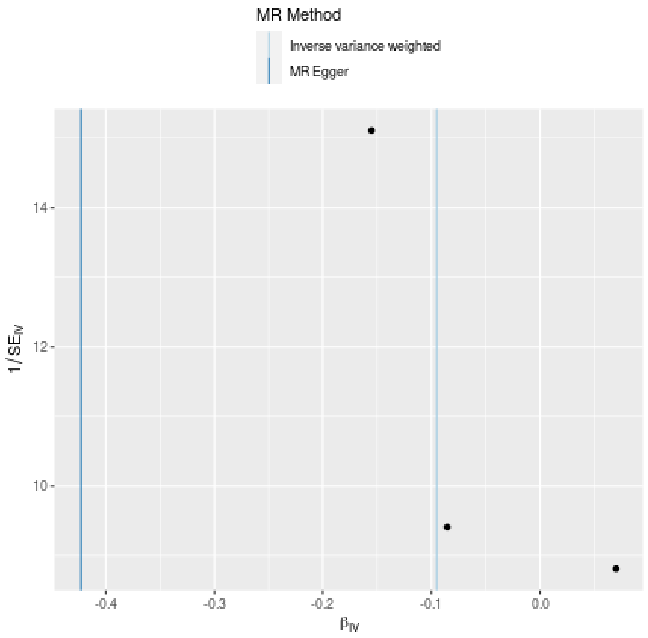	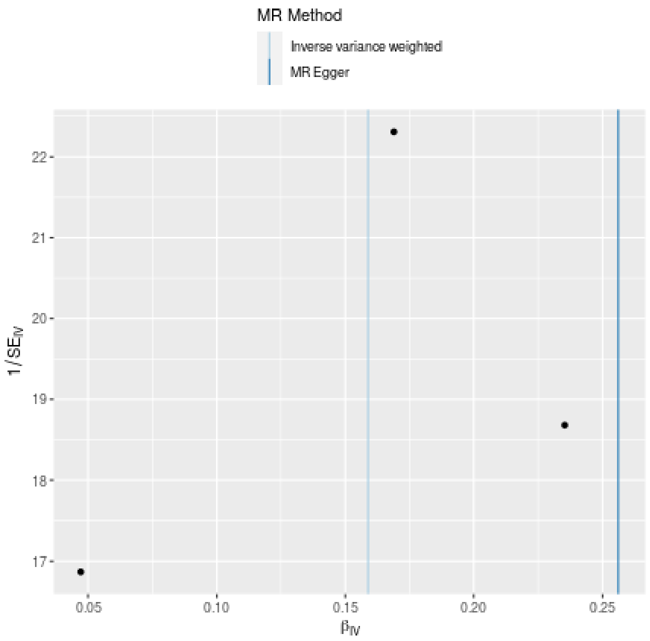

<table>
<tr><th>Tyrosine from Kettunen et al. 2016; n=24,925</th><th>Tyrosine from Locke et al. 2019; n=8,754</th></tr>
<tr><th colspan="2">Leave-one-out sensitivity analysis</th></tr>
<tr><td>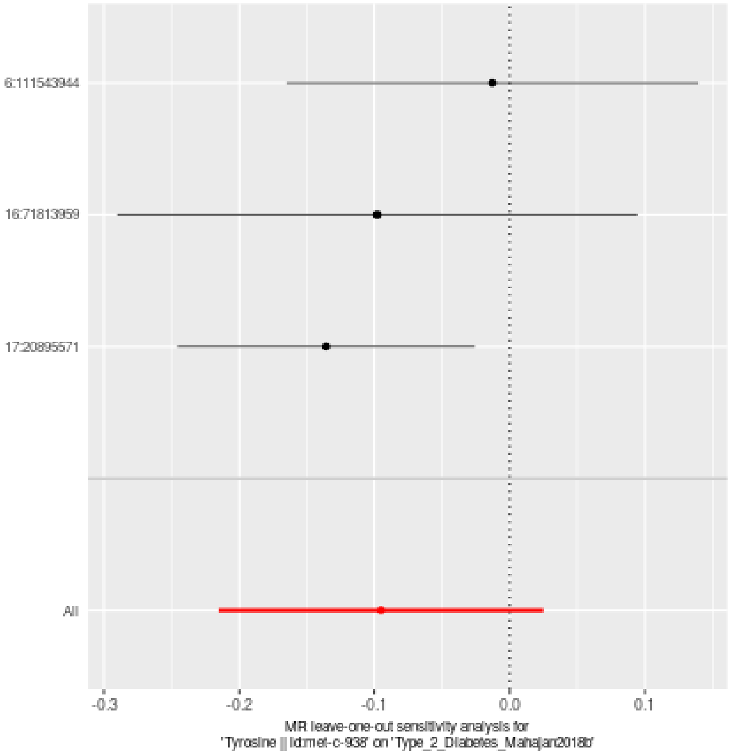
</td><td>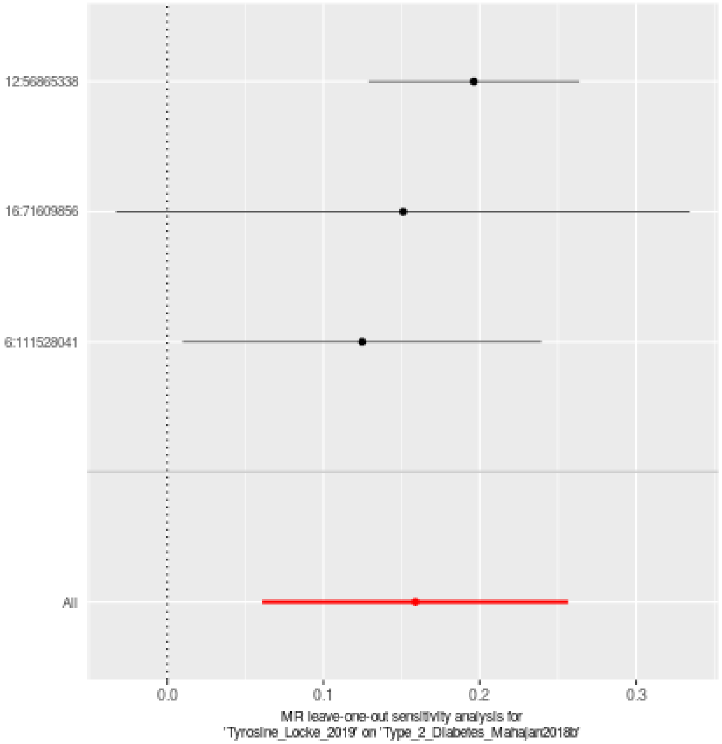
</td></tr>
</table>

Table S9 Associations of single genetic instruments used for MR of tyrosine from Kettunen et al. and Locke et al. and type 2 diabetes

See Excel file in Supplement B

Table S10 Total causal effects of tyrosine from Shin et al. and other risk factors for type 2 diabetes

Outcome	Data source*	MR-Base-ID/Author/PMID	Beta (SE) from IVW	P-Value	Heterogeneity Q-statistic, p-value	Directional horizontal pleiotropy** Egger-intercept (SE), p-value
BMI	UK Biobank	ukb-b-19953/ Ben Elsworth	-0.26 (0.33)	0.43	15.13, 0.001	-0.01 (0.05), 0.91
Waist circumference	UK Biobank	ukb-b-9405/ Ben Elsworth	-0.03 (0.25)	0.91	10.76, 0.005	0.0003 (0.04), 0.995
Fasting blood glucose	MAGIC	ebi-a-GCST000568/ Dupuis 2010/ 20081858	-0.39 (0.22)	0.08	1.58, 0.45	0.02 (0.03), 0.66
Fasting blood glucose adjusted for BMI	MAGIC	ebi-a-GCST005186/ Manning 2012/ 22581228	-0.68 (0.26)	0.008	3.91, 0.14	-0.02 (0.03), 0.58
Blood glucose	UK Biobank	ukb-d-30740_irnt/ Neale Lab	-0.43 (0.15)	0.004	0.06, 0.97	0.0007 (0.02), 0.97
Fasting blood insulin	MAGIC	ebi-a-GCST000571/ Dupuis J 2010/ 20081858	-0.16 (0.23)	0.50	1.14, 0.57	0.02 (0.03), 0.63
Fasting blood insulin adjusted for BMI	MAGIC	ebi-a-GCST005185/ Manning 2012/ 22581228	-0.01 (0.19)	0.95	0.73, 0.70	0.02 (0.02), 0.56

IVW, inverse variance weighted method; PMID, Pubmed identifier; SE, standard error;
Tyrosine was instrumented by three variants from Shin et al. [3] (see Table S3/S4)

*accessed via MR-Base platform [4]
** assessed by MR-Egger (>2 variants needed)

Table S11 Instruments from genetic risk score for insulin resistance*

SNP*	Chr:Position	Gene	Effect allele	Other allele	EAF**	Beta**	SE**	P-Value**
rs4846565	1:219722104	*LYPLAL1*	G	A	0.690	0.015	0.0026	2.01E-08
rs10195252	2:165513091	*GRB14*	C	T	0.442	-0.016	0.0026	4.87E-10
rs2943645	2:227099180	*IRS1*	C	T	0.372	-0.013	0.0025	1.37E-07
rs17036328	3:12390484	*PPARG*	T	C	0.903	0.015	0.0036	1.92E-05
rs6822892	4:157734675	*PDGFC*	A	C/G	0.646	0.011	0.0025	3.16E-05
rs3822072	4:89741269	*FAM13A1*	G	A	0.455	-0.0092	0.0024	1.19E-04
rs4865796	5:53272664	*ARL15*	A	G	0.708	0.015	0.0026	2.09E-08
rs459193	5:55806751	*ANKRD55/ MAP3K1*	G	A	0.783	0.014	0.0027	6.57E-08
rs2745353	6:127452935	*RSPO3*	T	C	0.550	0.014	0.0025	5.48E-09
rs731839	19:33899065	*PEPD*	A	G	0.659	-0.014	0.0026	1.72E-08

EAF, effect allele frequency; SE, standard error; T2DM, type 2 diabetes

* GRS for Insulin resistance [5]

**GWAS data from MAGIC on fasting insulin [6]

Table S12 Total causal effects of insulin resistance on amino acid traits from EPIC-Potsdam

Amino acid trait	N (SNPs)[a]	Beta (SE) from IVW	P-Value	Heterogeneity between SNPs; Q-statistic, p-value	Directional horizontal pleiotropy[b]; Egger-intercept (SE), p-value	Outlier detected
**Glycine**	9/10	-0.354 (0.756)	0.640	no; 7.57, 0.48	no; 0.04 (0.08), 0.65	no
**Glycine/Serine**	9/10	1.167 (0.756)	0.123	no; 5.06, 0.75	no; 0.12 (0.08), 0.12	no
**Phenylalanine**	9/10	0.155 (0.855)	0.856	no; 10.13, 0.26	no; -0.04 (0.09), 0.70	no
**Phenylalanine/Arginine**	9/10	1.592 (0.927)	0.086	no; 11.95, 0.15	no; 0.10 (0.09), 0.30	no
**Serine/Phenylalanine**	9/10	-1.895 (0.862)	**0.028**	no; 10.85, 0.21	no; -0.06 (0.09), 0.50	no
**Tryptophan/Glutamine**	9/10	0.080 (0.750)	0.915	no; 7.85, 0.45	no; -0.01 (0.08), 0.90	no
**Tyrosine/Tryptophan**	9/10	0.515 (0.748)	0.491	no; 4.66, 0.79	no; 0.03 (0.07), 0.71	no
**Tyrosine/Methionine**	9/10	1.486 (0.744)	**0.046**	no; 3.63, 0.89	no; -0.01 (0.07), 0.93	no
**xLeucine/Methionine**	9/10	1.626 (0.864)	0.060	no; 10.90, 0.21	no; -0.01 (0.09), 0.89	Yes (rs2943645)

	8/9	0.974 (0.783)	0.213	no; 4.31, 0.74	no; -0.052 (0.08), 0.49	no
**Valine/xLeucine**	9/10	-0.975 (0.916)	0.287	no; 11.84, 0.16	no; 0.04 (0.10), 0.72	no

IVW, inverse variance weighted method; SE, standard error; xLeu = Isoleucine + Leucine

[a] used instruments/suitable instruments available in the GWAS of amino acid trait; rs6822892 excluded as triallelic variant;

[b] assessed by MR-Egger (>2 variants needed)

Table S13 Associations of single genetic instruments used for MR of insulin resistance and amino acids traits from EPIC-Potsdam

See Excel file in Supplement B

Table S14 Total causal effects of insulin resistance and single amino acids

Amino acid trait*	N (SNPs)**	Beta (SE) from IVW method	P-Value	Heterogeneity; Q-statistic, p-value	Directional horizontal pleiotropy*** Egger-intercept (SE), p-value
EPIC-Potsdam					
Serine	9/10	-1.75 (0.75)	0.02	no; 7.54, 0.48	-0.091 (0.075), 0.22
Tyrosine	9/10	0.29 (0.81)	0.72	no; 9.38, 0.31	-0.016 (0.086), 0.85
Methionine	9/10	-0.96 (0.76)	0.21	no; 6.49, 0.59	-0.015 (0.075), 0.84
Shin et al [3]					
Serine	9/10	-0.07 (0.06)	0.21	no; 2.64, 0.95	-0.002 (0.006), 0.73
Phenylalanine	9/10	0.003 (0.03)	0.92	no; 4.64, 0.80	0.001 (0.003), 0.87
Tyrosine	9/10	0.05 (0.04)	0.20	no; 8.78, 0.36	0.001 (0.005), 0.88
Methionine	9/10	-0.02 (0.04)	0.65	no; 9.92, 0.27	0.003 (0.004), 0.53

IVW, inverse variance weighted method; SE, standard error

* single amino acids from ratios, showing causal influence by insulin resistance in EPIC-Potsdam

** used instruments/suitable instruments available in the GWAS of single amino acids; rs6822892 excluded as triallelic variant

Table S15 Associations of single genetic instruments used for MR of insulin resistance and single amino acids

See Excel file in Supplement B

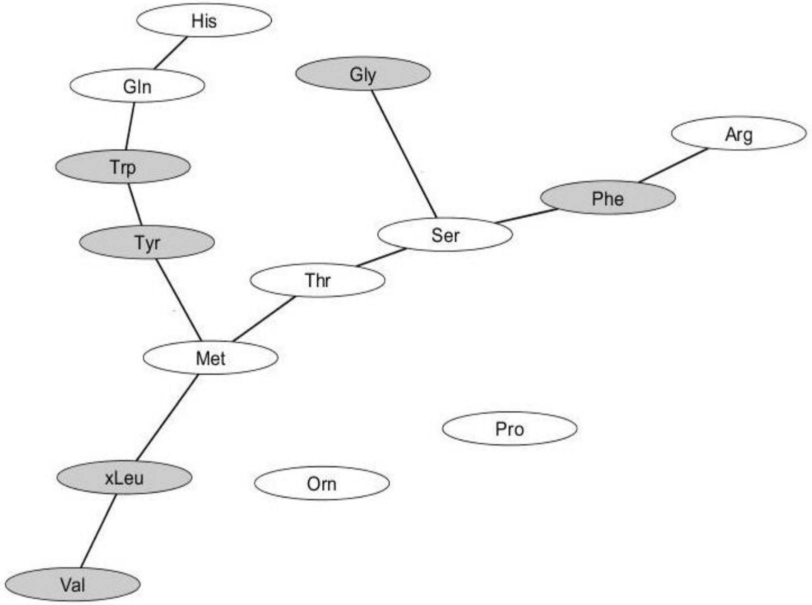

Amino acid traits tested within this study:

Glycine	Gly
Phenylalanine	Phe
Glycine/Serine	Gly/Ser
Serine/Phenylalanine	Ser/Phe
Phenylalanine/Arginine	Phe/Arg
Valine/xLeucine	Val/xLeu
xLeucine/Methionine	xLeu/Met
Tyrosine/Methionine	Tyr/Met
Tyrosine/Tryptophan	Tyr/Trp
Tryptophan/Glutamine	Trp/Glu

Figure S1 Network structure of amino acids within EPIC-Potsdam

Diabetes-associated amino acids are depicted in grey elipses; solid line indicates direct association between amino acids, GGM network was adapted from the publication by A. Floegel *et al*. [7] and drawn by using Cytoscape Software v3.2.1 [8]

xLeucine = Isoleucine + Leucine

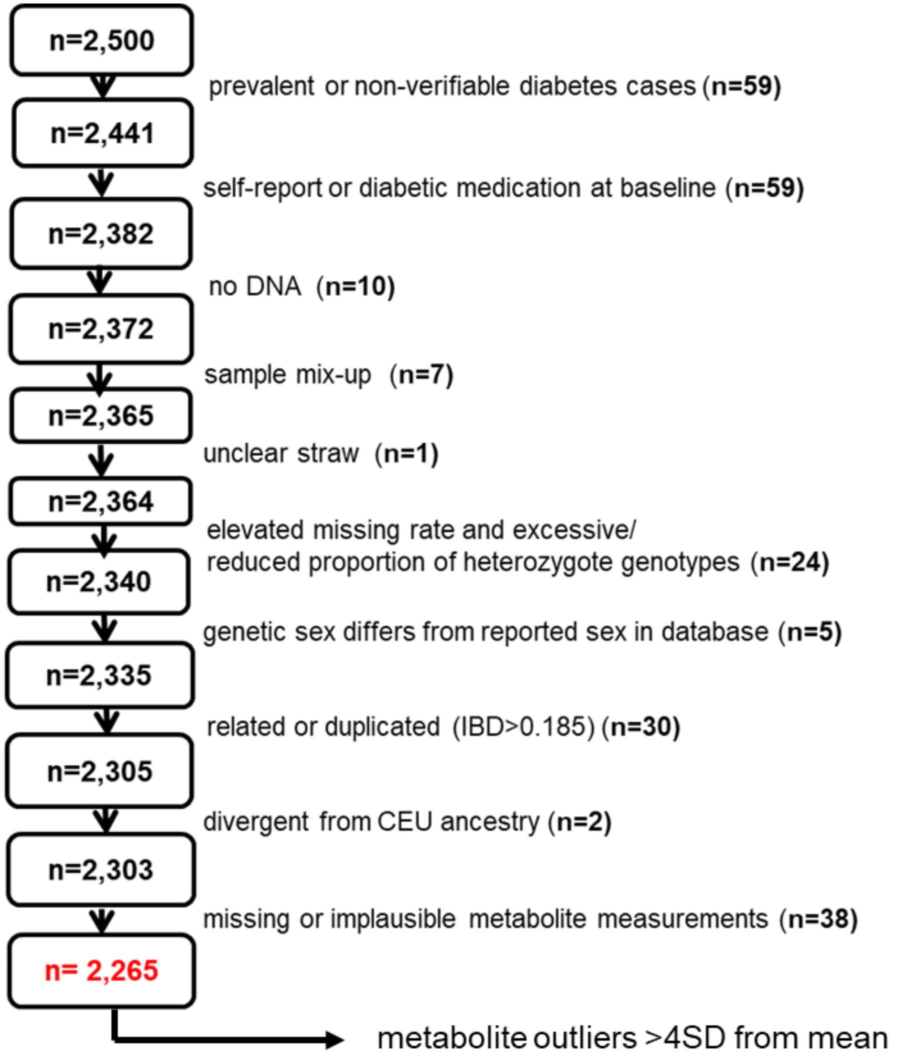

Figure S2 Flow-chart of final study population in EPIC-Potsdam

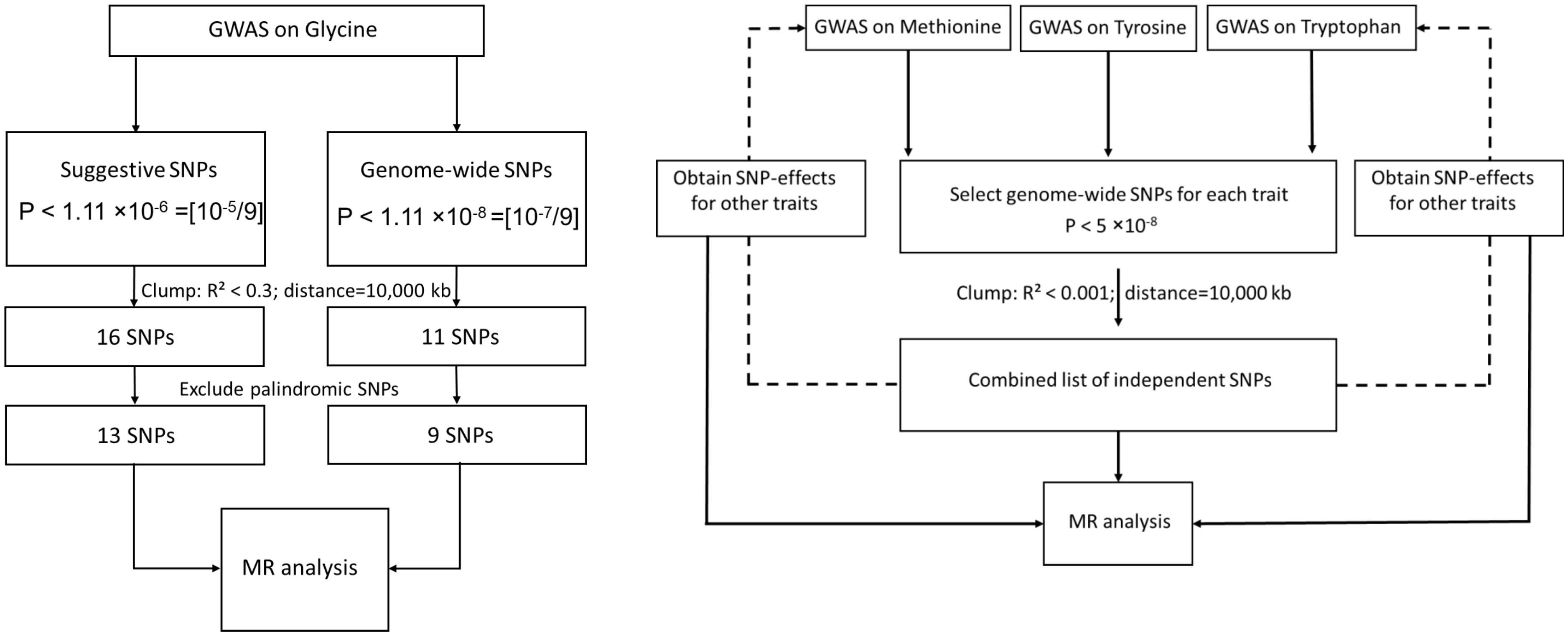

A **B**

Figure S3 Example flow charts of inclusion and exclusion of SNPs into the analysis

A univariable MR: First, we restricted to the set of SNPs according to the suggestive or genome-wide significance threshold. Next, we clumped the SNP list with thresholds $R^2 < 0.3$ or $R^2 < 0.001$ to receive independent instruments. **B** multivariable MR: First, we restricted to the set of SNPs according to the genome-wide significance threshold for each exposure. Next, we clumped the SNP list with thresholds $R^2 < 0.001$ to receive independent instruments and obtained associations with the other exposures. In MR analysis, palindromic SNPs were excluded.

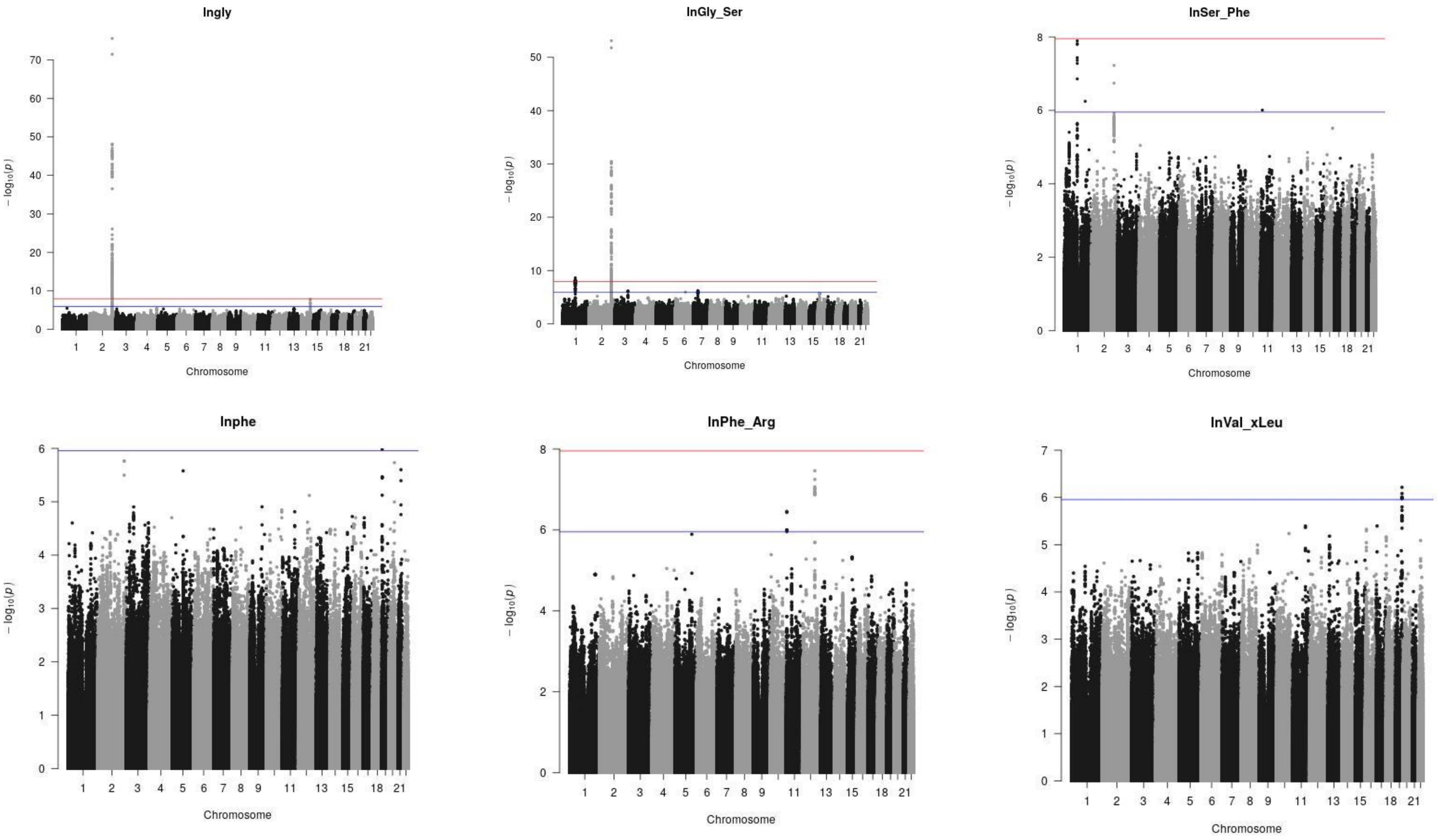

lngly
lnGly_Ser
lnSer_Phe
lnphe
lnPhe_Arg
lnVal_xLeu
$-\log_{10}(p)$
Chromosome

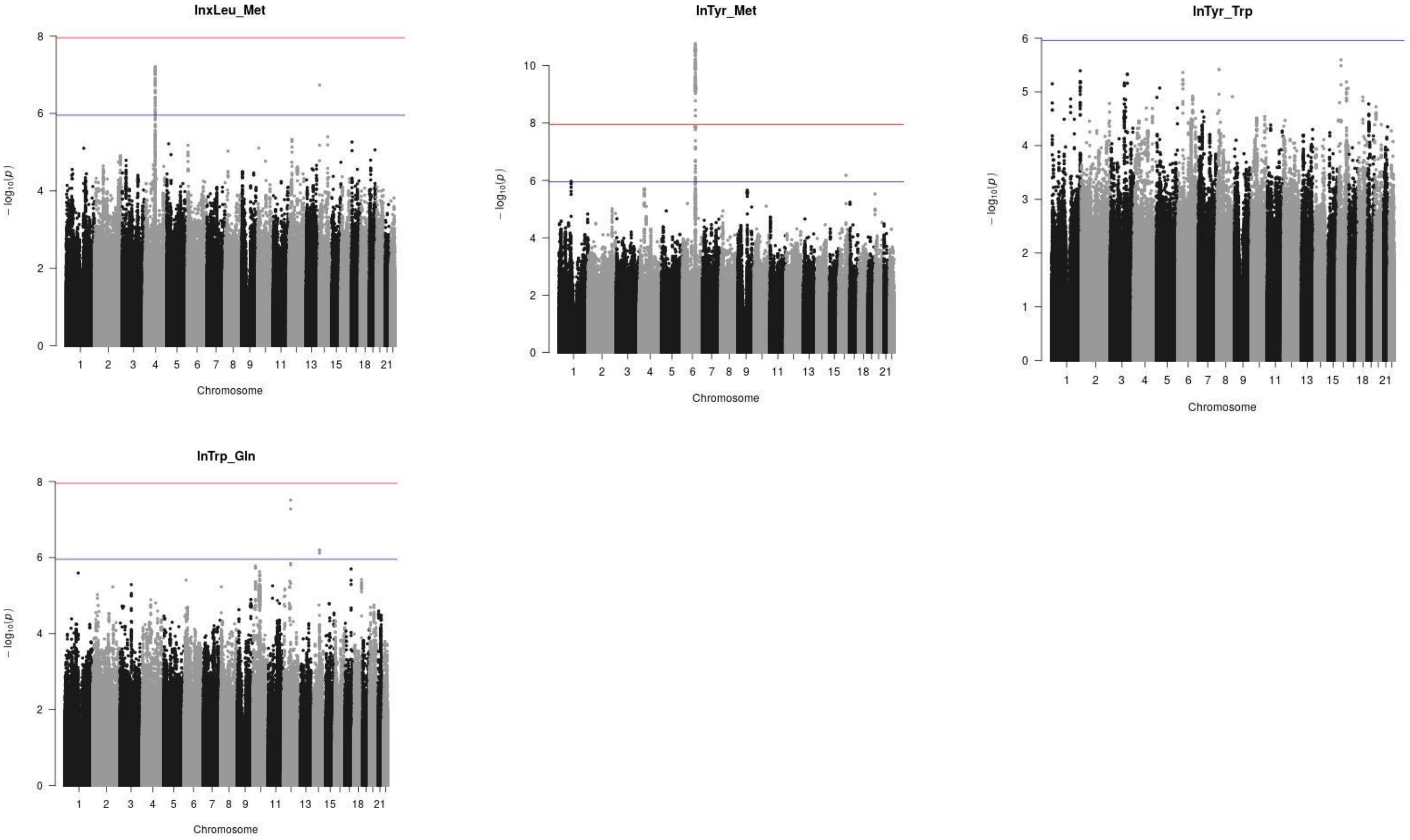

Figure S4 Manhattan plots visualizing GWAS results for amino acid traits in EPIC-Potsdam

Blue line indicates suggestive significance threshold ($P < 1.11 \times 10^{-6}$), orange line indicates genome-wide significance threshold ($P < 1.11 \times 10^{-8}$)

References

1. Mahajan, A.; Taliun, D.; Thurner, M.; Robertson, N.R.; Torres, J.M.; Rayner, N.W.; Steinthorsdottir, V.; Scott, R.A.; Grarup, N.; Cook, J.P., et al. Fine-mapping of an expanded set of type 2 diabetes loci to single-variant resolution using high-density imputation and islet-specific epigenome maps **2018**, 10.1101/245506, doi:10.1101/245506.
2. Mahajan, A.; Taliun, D.; Thurner, M.; Robertson, N.R.; Torres, J.M.; Rayner, N.W.; Payne, A.J.; Steinthorsdottir, V.; Scott, R.A.; Grarup, N., et al. Fine-mapping type 2 diabetes loci to single-variant resolution using high-density imputation and islet-specific epigenome maps. *Nat Genet* **2018**, *50*, 1505-1513, doi:10.1038/s41588-018-0241-6.
3. Shin, S.-Y.; Fauman, E.B.; Petersen, A.-K.; Krumsiek, J.; Santos, R.; Huang, J.; Arnold, M.; Erte, I.; Forgetta, V.; Yang, T.-P., et al. An atlas of genetic influences on human blood metabolites. *Nature Genetics* **2014**, *46*, 543-550, doi:10.1038/ng.2982.
4. Hemani, G.; Zheng, J.; Elsworth, B.; Wade, K.H.; Haberland, V.; Baird, D.; Laurin, C.; Burgess, S.; Bowden, J.; Langdon, R., et al. The MR-Base platform supports systematic causal inference across the human phenome. *Elife* **2018**, *7*, doi:10.7554/eLife.34408.
5. Scott, R.A.; Fall, T.; Pasko, D.; Barker, A.; Sharp, S.J.; Arriola, L.; Balkau, B.; Barricarte, A.; Barroso, I.; Boeing, H., et al. Common genetic variants highlight the role of insulin resistance and body fat distribution in type 2 diabetes, independent of obesity. *Diabetes* **2014**, *63*, 4378-4387, doi:10.2337/db14-0319.
6. Scott, R.A.; Lagou, V.; Welch, R.P.; Wheeler, E.; Montasser, M.E.; Luan, J.; Magi, R.; Strawbridge, R.J.; Rehnberg, E.; Gustafsson, S., et al. Large-scale association analyses identify new loci influencing glycemic traits and provide insight into the underlying biological pathways. *Nat Genet* **2012**, *44*, 991-1005, doi:10.1038/ng.2385.
7. Floegel, A.; Wientzek, A.; Bachlechner, U.; Jacobs, S.; Drogan, D.; Prehn, C.; Adamski, J.; Krumsiek, J.; Schulze, M.B.; Pischon, T., et al. Linking diet, physical activity, cardiorespiratory fitness and obesity to serum metabolite networks: findings from a population-based study. *International Journal of Obesity* **2014**, *38*, 1388-1396, doi:10.1038/ijo.2014.39.
8. Shannon, P.; Markiel, A.; Ozier, O.; Baliga, N.S.; Wang, J.T.; Ramage, D.; Amin, N.; Schwikowski, B.; Ideker, T. Cytoscape: a software environment for integrated models of biomolecular interaction networks. *Genome Res* **2003**, *13*, 2498-2504, doi:10.1101/gr.1239303.
